# “Something is clashing” – intentions to offer Medication-Free services within a traditional mental health ward

**DOI:** 10.3389/fpsyt.2026.1711274

**Published:** 2026-02-20

**Authors:** Lise S. Beyene, Marit H. Hem, Alain Topor, Mads S. Kopperud, Elin B. Strand

**Affiliations:** 1 Department of Public Health, Faculty of Health Science, University of Stavanger, Stavanger, Norway; 2 Department of Health Sciences, Institute of Nursing and Health, VID-Specialized University, Oslo, Norway; 3 Department of Psychosocial Health, Agder University, Kristiansand, Norway; 4 Department of Mental Health, Oslo University Hospital, Oslo, Norway; 5 Department of Psychiatry, Lovisenberg Hospital, Oslo, Norway; 6 Division of Medicine, Department of Digital Health Research, Oslo University Hospital, Oslo, Norway

**Keywords:** implementation, Medication-Free treatment, mental healthcare, milieu therapy, patient participation, psychosocial interventions, qualitative case study, recovery

## Abstract

**Introduction:**

Medication-Free treatment in mental healthcare aims to avoid pressure and coercion related to medication use, focusing on psychosocial interventions such as psychotherapy and milieu therapy. This approach emphasizes patient participation and voluntarism, aligning with human rights and shared decision-making principles. Knowledge about the challenges faced by mental healthcare professionals when introducing Medication-Free treatment in traditional mental health wards remains limited. This study aimed to explore the experiences of mental health professionals working in a ward that also offers Medication-Free services in order to gain a deeper understanding of this practice.

**Methods:**

A qualitative case study was conducted with two focus groups from a community mental health center ward in Eastern Norway. Data were analyzed using thematic analysis.

**Results:**

The overall theme, *Medication-Free services clash with a traditional medical system*, was a common thread among the three themes: 1) Working within an inflexible structure: Mental health professionals experienced frustration due to rigid procedures that limited their ability to tailor treatments to individual patient needs. 2) The relationship between power and responsibility is not well balanced: Mental health professionals perceived a significant imbalance in power dynamics, with doctors and psychologists holding most of the authority. 3) Relational work with patients does not receive sufficient focus: Mental health professionals felt that the emphasis on relational work was inadequate, impacting the effectiveness of Medication-Free treatment.

**Conclusion:**

Medication-Free services conflict with traditional medical systems due to fundamental differences in philosophy, structure, power dynamics and focus. Systemic changes are necessary to create a more flexible, empowering and relationship-focused milieu that supports both approaches effectively.

## Introduction

Medication-Free treatment in Norwegian mental healthcare refers to an approach that avoids pressure and coercion related to medication use, rather than being entirely free from all psychotropic medications. This treatment model aims to offer a holistic and patient-centered approach by focusing primarily on psychosocial interventions, such as psychotherapy and milieu therapy, without relying solely on psychotropic medications (1). This innovative approach seeks to provide therapeutic support based on patients’ active participation. Active patient participation in their own treatment is considered a human right (2) and is also regarded as important to the recovery process (3). Shared decision-making, which supports patient autonomy and self-determination, should be a fundamental aspect of mental healthcare (4). Patients may seek Medication-Free treatment due to negative experiences with medications and a desire for alternative options (5, 6). Research indicates that health outcomes in Medication-Free treatment units are comparable to those in traditional settings, with patients reporting higher satisfaction levels. This increased satisfaction may be attributed to the comprehensive psychosocial treatment programs that emphasize patient involvement without pressuring them to take medication (7).

In Norway, the authorities emphasized the importance of patients being able to choose between different treatments that involved Medication-Free courses, including establishing units for Medication-Free treatment, and in 2015, regional health authorities were tasked with establishing Medication-Free treatment within mental health care in close cooperation with user organizations. By 2018, 25 units offering Medication-Free treatment had been established in Norway (5). Following the establishment of these services, a debate emerged among professionals, user organizations and individuals with lived experience, focusing on ethical considerations, political governance versus evidence-based medicine, and the appropriateness of Medication-Free treatment for the most severely ill (8). A key question was whether biomedical knowledge or experiential knowledge should guide treatment. The knowledge base regarding the long-term use of antipsychotics in practice was also highlighted (9). A recent study comparing the outcomes of Medication-Free treatment and treatment as usual found that both treatment approaches led to significant improvement from admission to discharge. This suggests that clinicians can support patients in choosing the approach that best suits them (10).

Medication-Free treatment represents a shift towards more personalized and patient-empowered care. Focusing on non-medication-based therapies, it offers a viable alternative for individuals seeking different pathways to recovery. This aligns with the recovery perspective in mental health, which emphasizes personal empowerment, well-being, hope and the ability to live a fulfilling life despite mental health challenges. Recovery includes both clinical recovery, focusing on symptom remission and personal strengths, and personal recovery, prioritizing individual experiences, peer support and quality of life (11, 12). Medication-Free units emphasize psychosocial interventions and a holistic approach to mental health, including milieu therapy, which supports patients in making life changes and focusing on personal health promotion. This collaborative effort is intended to ensure that patients receive comprehensive care tailored to their individual needs. Practicing Medication-Free treatment involves multidisciplinary teams working closely with patients, requiring significant adjustments from traditional medication-based settings (13).

Medication-Free treatment is often implemented in mental health institutions based on a medical paradigm, which views mental disorders as illnesses requiring psychiatric expertise (8). Psychotropic medication is central in guidelines for psychosis treatment (14, 15) and is considered a key aspect of coercion (16, 17), which conflicts with the principles of Medication-Free treatment.

Implementing new treatment approaches can be challenging, with various factors affecting success. Braithwaite et al. (18) underscore that successful implementation of healthcare innovations depends on eight key factors: preparing for change, capacity for implementation (both people and setting), types of implementations, resources, leverage, sustainability, and desirable enabling features. Their systematic review highlights that healthcare settings are complex adaptive systems, where rigid, top-down strategies often fail. Instead, success in improving care quality and patient safety hinges on tailoring interventions to local contexts, fostering collaboration, and ensuring readiness for change. Another systematic review identified facilitators such as adapting the program to the local context, demonstrating early effects, and engaging both front-line clinicians and management. The most common barrier was resistance from front-line clinicians (19). Three aspects influence the adoption and implementation of new programs or treatments: the characteristics of the treatment itself, the patient, and the setting in which it will be implemented (18, 20–22). Factors such as perceived advantages, alignment with values, adaptability to the work situation, and ease of application are crucial (20). In a study interviewing mental health professionals (MHPs) about implementing an intervention to support the personal recovery of patients with a psychosis diagnosis, organizational readiness for change emerged as a key theme, highlighting the importance of developing MHPs’ skills and targeting both their intention to implement and their actual implementation behavior (23). In a qualitative study of MHPs, factors such as leadership support, clear goals, and leader engagement were emphasized as crucial for successfully maintaining the practice within the clinic (24). Successful implementation of recovery into mental health services requires flexibility, relationship building and incorporating lived experiences, with key factors including organizational commitment, addressing staff turnover, resource allocation and stakeholder engagement (25). However, the study of Hornik‐Lurie et al. (26) revealed that challenges remain, especially in acute wards and by MHPs, highlighting the need for ongoing training and active involvement from hospital administrations. A qualitative literature review revealed that limited knowledge and insufficient understanding of the recovery concept among MHPs constituted key barriers to implementation of recovery-oriented practice in mental health inpatient units (27). Reitan et al. (28) reported that MHPs working in a Medication-Free treatment unit emphasized recovery and therapeutic relationships over the mere absence of medication. Their approach prioritized patient autonomy, adequate time, stability and access to resources. Furthermore, Reitan et al. (6) highlighted that treatment in such units fosters patient empowerment, motivation, engagement in activities, and flexibility. These elements are shaped by the patients’ readiness and the therapeutic environment, underscoring the importance of empathic relationships and coherence in care delivery.

The success of Medication-Free services in mental healthcare is closely tied to the practices and engagement of MHPs. Effective care requires MHPs to support patients in making meaningful life changes, provide individualized attention, act as professional companions, and collaborate within multidisciplinary teams with a focus on personal health promotion (13). This approach demands that MHPs take on an active role and greater responsibility in the patient’s recovery process. Key to Medication-Free treatment is the relational competence of MHPs, which enables the development of positive, supportive relationships with both patients and colleagues. As described by Beyene et al. [(29) p.4], relational competence encompasses self-reflection, self-regulation, a genuine interest in understanding the patient, reciprocal engagement and the ability to make patients feel acknowledged. These qualities are essential for delivering high-quality, person-centered care to individuals facing mental health challenges.

There is limited understanding of the practical challenges MHPs encounter when implementing Medication-Free treatment within traditional mental health wards. This study aimed to explore the experiences of MHPs working in a ward that also offers Medication-Free services in order to gain deeper insight into this practice.

## Methods

A qualitative case study served as a suitable research design as we searched detailed, contextual understanding of the practice of a recently implemented service with the intention to be a Medication-Free treatment at a community mental health center. It enabled the exploration of this case’s essential features, meanings and implications (30, 31). Qualitative methods are essential for uncovering the unique and specific qualities of experiences of interventions in healthcare (32).

### Study context, case and participants

The context of this study was a ward at a community mental health center in central parts of Eastern Norway, which had been mandated to implement Medication-Free services two years before this study. The management of the community mental health center reached out to us to study the new practice they had implemented. The community mental health center serves a specific geographical region and specializes in treating mental health issues and substance use problems. The center comprises several wards, where the participating ward has an assessment and treatment service with 10 beds, four of which have been designated for Medication-Free treatment since 2018. Patients can be referred by a GP, specialist, or other regional treatment units, including the acute ward at the hospital, based on voluntary admission. In the community mental health center, psychiatrists and psychologists are in charge of the treatment. MHPs maintain the therapeutic milieu within the ward, operating on a three-part rotation. The ward is structured according to a medical model and organized as a psychosis treatment unit offering Medication-Free services, but also ensuring equitable treatment for all patients. Psychiatrists and psychologists, although primarily responsible for patient care, are organized under a separate unit. The service was further enhanced with the addition of a music therapist and a sports pedagogue. A clinical psychologist served as the Medication-Free program coordinator, responsible for holding regular meetings with service providers at the unit, informing other parts of the treatment system about the service, maintaining connections with similar units across the country, and, together with the leadership and intake team, evaluating requests and referrals for Medication-Free treatment. By offering Medication-Free services, they have added extra resources that benefit all patients. The only difference for patients on Medication-Free treatment is that they are tapering off the psychotropic medication, which may take up to three months, and the standard is that cognitive therapy should replace medication. All patients are offered a safe and predictable milieu, conversations, to work with a sports pedagogue and music therapist, and they can be admitted for up to three months. The frame is set so there will be no follow-up after the three months of admission.

Participants for this study were recruited by the head of the department, with inclusion criteria focused on MHPs experienced in working at the ward that offered Medication-Free services. The 10 MHPs participating in this study were aged from 31 to 60 years. They consisted of two males and eight females who had from nine months up to 38 years of experience in mental care inpatient settings. The study participants comprised five registered nurses, four registered mental health nurses, and one auxiliary nurse specialized in mental healthcare. One of the authors (MK) worked as a psychological specialist at the same department where the MHPs worked at the time the interviews took place, but all participants were unknown to the interviewers (MHH and EBS).

### Data collection

The data collection involved two distinct focus group interviews with five participants in each group. Focus group interviews, facilitated by group reflections and discussions, yield valuable data on the experiences and viewpoints of the stakeholders (33). The interviews were conducted at the community mental health center in June 2020 by MHH and EBS. Each session lasted for approximately 60 minutes. The central theme developed from the focus group conversations centered on participants’ experiences of offering Medication-Free services in inpatient mental health care. Throughout the discussions, they shared insights into their everyday practice settings and explored challenges and possibilities they encountered. All interviews were audio-recorded, transcribed, treated confidentially and securely stored (34).

### Analysis

A thematic analysis of the qualitative data was conducted, drawing inspiration from Braun and Clarke (35, 36). The goal was to identify patterns and themes in the collected data relevant to understanding the experiences of MHPs working in an inpatient Medication-Free service. The analysis involved six steps, moving back and forth between the various steps: Firstly, the authors read transcribed interviews multiple times to become acquainted with the data. Secondly, initial codes were created and organized into groups. For instance, one code was related to ‘patients rarely fit into the milieu’. In step three, patterns were identified as similarities and differences between codes (e.g., ‘the ward’s structure must be followed’). The abstraction was then validated in step four by checking if themes reflected the data and patterns. In step five, themes were refined and named (e.g., ‘dealing with a restricted structure’). While each specific theme is essential, it alone does not fully contribute to a comprehensive understanding of the experiences of MHPs working in an inpatient Medication-Free service. In the final step, the authors interpreted the patterns and identified the overall theme.

### Ethical considerations

The study adhered to the provisions of the Declaration of Helsinki (34), which entails fundamental ethical principles such as informed consent, privacy rights, and respect for personal integrity and dignity (37). All participants provided informed consent after receiving both written and oral information about the project. The text maintains participants’ anonymity. The research project’s protocol received approval from the Norwegian Social Science Data Service (NSD), which assessed privacy protection aspects (approved on November 14, 2019). Since the study did not involve patients as participants, Norwegian regulations did not require approval from the Regional Committee for Medical and Health Research Ethics.

## Results

The aim of this study was to explore the experiences of MHPs working in a mental health ward intended to offer Medication-Free services. The overall theme *Medication-Free services clash with a traditional medical system* was developed as it appeared as a common thread in the themes, *Working within an inflexible structure; The relationship between power and responsibility is not well-balanced*, and *Relational work with patients does not receive sufficient focus* (**Table 1**).

**Table 1:** Overview of the results.

Overall theme: Medication-Free services clash with a traditional medical system			
Themes	1. Working within an inflexible structure	2. The relationship between power and responsibility is not well-balanced	3. Relational work with patients does not receive sufficient focus
Subthemes	The ward’s treatment plan must be followed	Lack of interdisciplinary cooperation	Challenging emotions are barriers to engaging with patients
	Diagnosis and medication are central	Lack of ownership of the treatment	Time limitations in relational work
	Lack of strategy	Feeling powerless and unimportant	The challenge of dialoguing and involvement

### Overall theme: Medication-Free services clash with a traditional medical system

The overall theme summarizes the experiences of MHPs working in an inpatient mental health ward intended to also offer Medication-Free services, highlighting the challenges and dynamics the participants face. The participants in this study expressed experiences of a rigidity of structures, power imbalances between the professionals, and insufficient focus on relational work, which seemed to upset them. They found it challenging to adapt to individual patient needs, which is essential for Medication-Free services, and they experienced failure in applying Medication-Free treatment. The participants expressed having challenges getting their knowledge and practice recognized by the doctors and psychologists. They felt undervalued and saw themselves as victims of a system that did not provide adequate resources, leading to feelings of helplessness and a reliance on external support to bring about necessary changes. This disharmony underscores the gap between their professional aspirations and the realities of their working environment. One participant stated:

*I feel a bit of the problem is that you try to implement Medication-Free treatment into a system that is built on medication. Something is clashing.* (Kristian)

The Medication-Free service, which emphasizes patient empowerment and holistic care, does not appear to align with the rigid structures and hierarchical nature of traditional medical systems that the participants were part of.

### Theme 1: Working within an inflexible structure

This first theme describes the participants’ experiences of working within an inflexible structure that is not ideal when intending to provide Medication-Free services, which made them feel frustrated. They often encountered rigid procedures that limited their ability to tailor treatments to individual patient needs. This lack of flexibility not only hindered their professional autonomy but also led to a sense of helplessness and dissatisfaction, as they were unable to fully implement therapeutic approaches. The participants expressed that these constraints negatively impacted their job satisfaction and the overall effectiveness of the care they provided. The three sub-themes describe this theme in more detail: *The ward’s treatment plan must be followed*; *Diagnoses and medication are central; Lack of strategy*.

#### The ward’s treatment plan must be followed

The participants explained that not all patients in the ward are on Medication-Free treatment, but all patients were to be treated the same way. Furthermore, the participants shared experiences of patients rarely fitting into the milieu. They conveyed that the patients have varying opinions about the therapy, and they cannot accommodate all patients’ expectations. One participant expressed it like this:

*We set the goal, and then the patient just has to adapt to the goal in a way … It doesn’t feel like we have a joint project with the patient*. (Truls)

They experienced that most patients needed more time for tapering off psychotropic medication, but they were to follow the tapering routines that were set. As MHPs, they experienced a lack of person-centered arrangements for the patients, and the emphasis on patients developing new coping strategies was low. From what the participants shared, we understand that they are expected to adhere to the ward’s structure, even when they register that not all patients follow up on their treatment plan. Some participants understood this as a sign of lack of motivation, some as a sign of laziness, while others explained this by the fact that they do not have an arrangement that is helpful for everyone. One participant conveyed that if the patients do not fit into the ward’s structure, they cannot be kept in the ward:

*We lack the necessary tools if we’re not using medication. Ideally, we should be able to protect people, but we can’t do it unless they cooperate. If they do, we can manage it, but then they need to be isolated in their own room and make an agreement.* (Eline)

The participants expressed that they wished for other interventions and extended treatments for the patients admitted for Medication-Free services to offer the patients optimal treatment. They believed that they were dependent on the medical plan formulated by the persons in charge of treatment, and this plan did not give them the flexibility to contribute to other approaches. By stating that they leaned solely toward the medical plan, they revealed that they would not take responsibility for finding a way to practice their expertise because they believed that the doctors and psychologists had this responsibility.

#### Diagnosis and medication are central

Throughout both focus group interviews, the participants had a high focus on diagnosis and medication, elaborating more on the various diagnoses patients had and how much medication they took, than on the Medication-Free service they were to offer. They had a great focus on medication to help their patients. One of the participants said:

*The goal is sort of to get rid of the medication, versus finding a way to live without medication. The focus is only on which symptoms and how the symptoms are developing, which occur because we take the medication away, versus why they took the medication in the first place and how they can learn to cope with their problems.* (Truls)

The participants were convinced that the diagnosis was to determine what treatment the patients needed. One participant expressed that:

*I don’t believe that if you have a severe diagnosis and have been on medication for so many years, you can taper off and stop taking medication*. (Vera)

This participant expressed little knowledge about Medication-Free treatment. She conveys that she does not believe in the treatment program and is opposed to it. The participants had little focus on how they could help the patients with symptom reduction and coping with daily life.

#### Lack of strategy

The participants expressed that they did not know how to offer Medication-Free services. Even though they had been offered some resources and time to facilitate the treatment, they lacked experience and knowledge about how to facilitate Medication-Free services. They clearly stated that no treatment strategy was in place:

*It was planned that they should receive structured cognitive therapy and such, but it has not been fully initiated yet … I feel like we’ve got a machine, but nobody quite knows how to operate the machine*. (Kristian)

The participants expressed that they had been little aware of treating patients on Medication-Free treatment differently from other patients:

*I haven’t properly heard anything about it [Medication-Free treatment] as long as I’ve worked in this ward. I have only heard that the treatment is not quite set … That something is missing.* (Marthe)

The participants wished for consistent follow-up from the same healthcare providers over time. They recognized the need for a structure that caters to patients’ needs and a treatment plan that differs from the usual medical approach. Although they understood the importance of having a well-organized treatment plan for quality care, they felt unable to act on it.

### Theme 2: The relationship between power and responsibility is not well-balanced

The second theme we have identified was that participants experience the relationship between power and responsibility between MHPs and doctors and psychologists as unbalanced. MHPs perceived that doctors and psychologists were the ones who hold the power in the system and missed them taking the responsibility that comes with that power, while at the same time resigning from their own responsibilities. The three sub-themes describe this theme in more detail: *Lack of interdisciplinary cooperation; Lack of ownership of the treatment; Feeling powerless and unimportant*.

#### Lack of interdisciplinary cooperation

The participants experienced that the different professional groups relied on their professional perspectives rather than seeking cooperation between them. The interviews revealed that the MHPs experienced that the doctors and psychologists were not interested in their perspective:

*There’s something about the dialogue between us and the doctors and psychologists. It has something to do with listening, to take what we say seriously*. (Eva)

This means that the doctors and psychologists, as our participants experience it, do not focus sufficiently on milieu therapy and that they do not know what is happening at the ward:

*Those who admit patients and those who make judgments about who can be included in the Medication-Free service are people who don’t work in the milieu. There is a completely different assessment from those sitting in the conversation room than those working in the milieu.* (Truls)

This means that there was little talk about interdisciplinary cooperation and that those who do the assessments related to patient treatment are those who do not work at the ward “in the milieu”. MHPs wanted the doctors and psychologists to be “more involved” and they generally wanted more attention to milieu therapy since there were no structured milieu-therapeutic arrangements. They believed it would have been easier to make adequate decisions if the doctors, psychologists and the MHPs worked together as a group. The following dialogue took place between three of the participants:

Marthe: *We’ve talked about having two or three doctors and psychologists who work closer to us. I think we would form a team so that we could work closer and maybe make plans for the milieu therapy*.

Vera: *It would have been a great advantage to have the same doctors and psychologists working with us*.

Eva: *I think that is very important. Really. It has to do with continuity.*

Additionally, the fact that MHPs thought that there would be better milieu therapeutic arrangements if they had more doctors and psychologists on the team means that a closer team collaboration would also ensure that patients would be more involved in the treatment. For example, MHPs believed that because there is little dialogue with patients about reducing medication, the medication reduction sometimes becomes too fast.

In addition, in connection with the establishment of Medication-Free treatment, two new professional groups were employed, namely, a sports pedagogue and a music therapist. This seemed to the participants to be a reasonable measure and meant possibilities for more interdisciplinary cooperation. However, this has not happened. They said that the sports pedagogue and music therapist are present, but that they do not participate in the team meetings. They realized that sports and music therapists have a different focus than MHPs since they are not primarily concerned with disease and medicine. The problem is, according to the participants, that they do not cooperate.

The lack of interdisciplinary cooperation can be summarized as Ingrid formulated it:

*It may not feel like you have a common project with the patient. But the music therapist has one project, the sports pedagogue has one, the psychologist has a project, we also have a project in a way, the patient may have a completely different project … You fight about how to get the space and time to run your project.*

However, the participants did not mention anything about whether they took responsibility for collaborating with their colleagues. This may illustrate MHPs’ own powerlessness and that *the relationship between power and responsibility is not well-balanced.*

#### Lack of ownership of the treatment

The participants assumed that no one with authority had ownership of the Medication-Free treatment scheme. There is no one with ‘professional authority’, which means having expertise in reducing medication, who can be the driving force, Eva said. She assumed that the therapists feel that the offer is “*pulled down over their heads*”, and then they “*don’t quite know what to offer*”. The MHPs believed that Medication-Free treatment options “*belong to the management*”. The participants expressed frustration and confusion due to the management’s failure to clearly communicate and integrate the offer throughout the organization.

As a consequence of insufficient communication, the participants experienced that the cooperation between therapists and MHPs was problematic, and that the patients ended up as “*losers*”, meaning that they did not receive the expected services.

Another situation that was unfortunate for the patients concerned was that there were no solid plans for a stay with Medication-Free treatment. They described that a long-term plan that extends beyond a time-limited stay would be important. They said that some patients who have been given a plan to taper off over a few weeks have become so unwell that they have been admitted to the emergency department. This led to a “*vicious circle*”.

The participants further said that there should be a focus on milieu therapy when patients are reducing medication. MHPs were concerned that the patients have “*underlying problems*” and that these must be seen in connection with the use of medication: “*How can we help him to live without all those pills?*”, they asked. When it comes to working with milieu therapy, Truls believed that it is about being able to “*do things together with the patient and … be of help to them*”. He said that he missed more space for milieu-therapy, such as being with the patients “*so they get out of bed and out of the room*”.

#### Feeling powerless and unimportant

The participants thought that the psychologists and the doctors make little use of the observations from the MHPs or that “*they* [MHPs] *are not taken seriously enough*” (Marthe). Eline said that “*no matter what we say, it does not help*”. They said that only the doctors and psychologists are present in the management group, which means that the MHPs are not part of the discussions and decisions, and consequently, there cannot be an open dialogue between doctors and psychologists, and the MHPs:

*… We may at times have to argue with the doctor and the psychologist. It is more than once I have been afraid that we might push a patient so much that we are anxious about the patient acting out, for example. Because we will be short of help, yes, that it will be too late, then, or that they could perhaps have avoided some emergency admissions, for example, if we had managed to help earlier.* (Ingrid)

This is another example of how the imbalance between power and responsibility may negatively impact the patient. Furthermore, participants were more likely to notice when patients’ conditions worsen compared to doctors and psychologists, as they interact with the patients constantly.

### Theme 3: Relational work with patients does not receive sufficient focus

The third theme deals with the MHPs’ thoughts and experiences regarding the treatment process, barriers to engaging with patients, fear of failure, and availability of therapists. The three sub-themes describe this theme in more detail: *Challenging emotions are barriers to engaging with patients; Time limitations in relational work; The challenge of dialoguing and involvement.*

#### Challenging emotions are barriers to engaging with patients

Participants agree that rapid medication tapering heightens failure risks, potentially worsening patient conditions and leading to stress and a sense of failure for MHPs. Open wards struggle with aggressive or very unwell patients, potentially necessitating transfers to acute care, which MHPs view as setbacks for the patients. The participants also emphasized that the ward, being an open one, is not equipped to handle aggressive or severely ill patients.

*I’ve seen it in some cases where the goal is to reduce medication, and then you taper down, taper down, and suddenly the patient doesn’t do well or becomes very ill. Since we’re an open ward, we can’t handle aggression, so it often results in a transfer to acute care, which is a huge defeat for many. Then, it seems impossible to stop using medication.* (Kristian)

The participants expressed concern about patients getting worse, which may lead to frustration and doubt in the Medication-Free process. MHPs feel inadequately prepared to manage strong emotional reactions, creating barriers to engaging with patients and impacting the success of Medication-Free offers. Trying to prevent ending up in a situation feeling discomfort associated with lack of competence or failure may be one of the barriers that prevent engagement in and trying to achieve a good Medication-Free offer for patients.

The participants focused mainly on symptom fluctuations and management during tapering but stressed the need for engagement and being closely involved with patients’ emotions and reactions. They acknowledged feeling unprepared to handle various patient reactions post-tapering. At the same time, as it appears above, they do not feel competent to cope with all kinds of reactions and feelings that patients experience in the wake of the tapering.

#### Time limitations in relational work

Failed medication tapering is often attributed to poor communication between the MHPs and the psychiatrist, and impatience and time constraints seem to be the most important contributing factors of failure. The participants mentioned that the pressure of rapid tapering in a short-stay framework may exacerbate impatience. Additionally, pressure from the patient who wants the tapering process to happen quickly makes it challenging for the doctors to hold back. When the tapering process is too fast, the patient does not have time to adjust to a reduced medication dose, neither to manage the emotional reactions that may arise, nor learn alternative ways to cope with their situation or reactions other than using medication.

*But I see with the doctors, when we have patients with substance use problems or heavy use of benzodiazepines, they taper down too quickly as well, without the patient having time to adjust and find … other coping strategies.* (Trine)

It also appears that the doctors are impatient and rush tapering, leaving patients without supervision for handling side effects of tapering or new ways of coping with a life without medication. It looks as if the MHPs, who are supposed to follow up the patients during tapering, have no control in this process.

The participants recalled positive experiences aiding patients’ medication management, emphasizing patient coping skills to manage daily life challenges. This might involve being able to care for their children, go shopping, or attend treatment appointments. The MHPs pointed out that while the patients are admitted to the ward, they contribute with constructive input and usually focus on and assist the patients with practical challenges for handling everyday life at home, and that this is important.

There was agreement that aiming for a total recovery is too high a goal, and the participants stressed the value of accepting personal challenges in the recovery process as important.

*I think a lot of it is about not thinking in black and white. Like, maybe I can take some medication, but I can manage on much less. I do other things. Accepting that you have some extra challenges in daily life that others may not have. Those who can accept that manage quite well. But those who think, “Either I’m healthy, or I’m sick, and then either I take medication or I don’t”, they struggle more with managing life because they set goals that are too difficult to achieve*. (Eline)

In this context, acceptance can also mean recognizing that medication might help the patient become more open and easier to connect with in conversations with therapists and MHPs. When patients are very unwell, medication can make them more available for other therapeutic work:

*Especially when they’re very ill or acutely getting worse, medication can help shift them into a state where they’re more accessible to work with in other ways and dare to make changes.* (Trine)

Tapering and recovery are time-consuming and need ongoing support, yet institutional constraints hinder this. Despite additional resources, time for patient interaction remains limited due to administrative duties. In this context, MHPs are constrained by limited lengths of stay, lack of resources, and structural conditions that hinder effective follow-up after discharge. When the framework within which MHPs operate is insufficient to support successful medication tapering, as described by participants, it introduces yet again a barrier to progress, namely insufficient time.

#### The challenge of dialoguing and involvement

The participants saw patient dialogue as crucial for preparing for tapering challenges, yet this dialogue is often lacking.

*There’s also a lack of dialogue with patients who are tapering off medication. You need to have a strong relationship to talk about difficult things, like what to do if the patient experiences worsening symptoms during the tapering process. (…) But there’s no dialogue aboutt that.* (Kristian)

MHPs understand the importance of availability and contact with patients, but report insufficient dialogue. The way they talk about it makes it sound as if they do not implement this in their own practice and that the responsibility for such a dialogue lies elsewhere than with themselves and in their role. Additional staff for logistical duties to handle tasks such as cooking and other practical duties have been hired along with the music therapist and the sports pedagogue who conduct daily activities with the patients. However, daily activities for the patients primarily took place with the music therapist and sports pedagogue. The MHPs seem less involved and somewhat resigned, as if they have outsourced the daily activities and therapeutic work to the music therapist and sports pedagogue.

*I think it’s important from a therapeutic perspective that patients have something to engage in during the day, like activities. But we don’t have many options, so it often just comes down to sports and music therapy, and then people just end up sitting around waiting. There’s no plan around that.* (Vera)

The participants experienced that patients’ tendencies to withdraw impacted MHPs’ commitment to relational work. Some patients are eager to taper off medication, but resistant to taking part in activities. Low motivation among the patients seems to affect the MHPs’ commitment to relational work:

*Sometimes it’s quite demotivating to get people involved in daily activities.* (Eline)

## Discussion

The present study examines the experiences of MHPs working in a mental health ward intended to offer Medication-Free services for some patients. The results indicate that Medication-Free services clash with a traditional medical system. This entails that the MHPs are working within an inflexible structure, the relationship between power and responsibility is not well balanced, and the relational work with patients does not receive sufficient focus. We will discuss challenges and limitations of the planned Medication-Free service in light of existing research, aiming to contextualize these issues within the broader body of knowledge on the topic.

### The medical context for Medication-Free services

According to Braithwaite et al. (18), implementation outcomes are deeply influenced by the context in which they are carried out. The results of this study show that the implementation of Medication-Free services within a traditional medical context presents substantial challenges. A central tension lies in the attempt to practice Medication-Free treatment in a context which is fundamentally structured around medical interventions. A traditional medical system typically focuses on diagnosing and treating mental health disorders primarily through medication, fostering rigid procedures, standardized treatment plans, and a hierarchical structure where medical professionals make most decisions. In contrast, Medication-Free services advocate for a holistic approach that emphasizes personal health promotion and therapeutic support without relying on psychotropic medication (13). This approach requires flexibility, adaptability and shared decision-making (25), which can be stifled by the protocol-driven nature of traditional systems. Additionally, the power dynamics in traditional settings often leave MHPs with significant responsibilities but limited authority, further complicating the integration of Medication-Free services. The results of this study show a hierarchical structure where doctors and psychologists hold decision-making authority, which marginalizes the contributions of other MHPs. This power imbalance not only devalues their expertise but also limits their ability to advocate for or implement alternative therapeutic approaches. Braithwaite et al. (18) highlight the importance of distributed leadership and collaborative practice in facilitating change, conditions that appear lacking in this study. These fundamental differences in treatment philosophy create a misalignment that makes it difficult to effectively implement and sustain Medication-Free services within the traditional medical context. For Medication-Free services to be successfully integrated, systemic changes are needed, both in organizational structures and in the professional culture of mental health care (20, 21), toward a context grounded in humanistic values and principles (13).

### How mental health professionals engage with Medication-Free services

The capacity of MHPs to develop Medication-Free services is shaped not only by structural and procedural factors but also by deeply human and relational dimensions (29). These include professional skills, attitudes, interprofessional cooperation, and leadership factors that are key to successful implementation, as highlighted in implementation science literature (18).

Our study shows that MHPs felt unprepared and unsupported in the implementation of Medication-Free services. The participants express uncertainty about their roles, limited knowledge of psychosocial alternatives, insufficient training and experience, and a lack of ownership over the treatment process. These challenges reflect a broader issue of inadequate training and misalignment within organizational structures. Leadership and team development are therefore essential to equip the staff with the competencies required to engage in Medication-Free services and to foster a shared understanding of the goals and values underpinning Medication-Free treatment (23, 25, 27). This aligns with Leamy et al. (23), who emphasize that both the intention to implement and actual implementation behavior must be actively supported through competence development. In the absence of such development, MHPs may experience heightened insecurity, particularly when patients exhibit strong emotional reactions or worsening symptoms during medication tapering. This echoes Greenhalgh et al. (20), who argue that perceived complexity, lack of compatibility with existing work practices, and insufficient support structures significantly reduce the likelihood of successful adoption.

Psychological factors such as MHPs’ attitudes, beliefs and the influence of professional norms significantly affect the adoption of new practices. In our study, some MHPs expressed skepticism about the feasibility of Medication-Free treatment, particularly for patients with severe diagnoses. These attitudes may stem from entrenched biomedical paradigms and highlight the need for reflective practice and value-based dialogue across professional groups (20). As Braithwaite et al. (18) emphasize, implementation efforts should be tailored to the needs and perspectives of those involved. Customizing the implementation process to different stakeholders, such as clinicians, managers and patients, can promote more positive attitudes and greater engagement.

High levels of cooperation between managers and clinicians have been shown to facilitate the uptake and spread of new practices (19). However, our findings suggest that such cooperation was lacking. MHPs reported limited interdisciplinary collaboration and a perceived disconnect between frontline staff and decision-makers. This disconnect may also explain why clinicians involved in direct practice often rate the impact of implementation efforts on clinical care more highly than do policymakers (18). When implementation strategies are designed without sufficient input from those delivering care, they risk being perceived as top-down mandates rather than collaborative innovations.

Leadership plays a pivotal role in bridging these gaps. Effective leaders not only provide strategic direction but also foster inclusive environments where all professional voices are heard and valued. In our study, the absence of clear leadership and strategic coordination contributed to confusion and fragmentation. Strengthening leadership at both the managerial and clinical levels is therefore crucial for building capacity and sustaining change (19, 20).

In summary, enhancing MHPs’ capacity to deliver Medication-Free services requires a people-centered implementation strategy. This includes targeted training, supportive leadership, interdisciplinary collaboration and a commitment to tailoring implementation efforts to the needs and values of all stakeholders. Only by addressing these human factors can organizations move beyond structural reforms and realize the transformative potential of Medication-Free mental health care.

### Resources to practice Medication-Free services

This study indicates that insufficient resources, both structural and interpersonal, constitute a major barrier to implementing Medication-Free services. Despite strong ideological alignment with recovery-oriented care, the lack of time, training, interdisciplinary collaboration, and clearly defined professional ownership undermines the MHPs’ ability to deliver Medication-Free services effectively. This echoes findings from Stone et al. (19), who identify lack of time and resistance among front-line clinicians as the most common obstacles to successful implementation.

Relational continuity and therapeutic availability are well-documented prerequisites for Medication-Free services (13, 28), and this was confirmed in this study. MHPs reported that time constraints and short stays, limited to a maximum of three months with no follow-up, made it difficult to build therapeutic alliances or work through emotional challenges and behavioral change. These conditions conflict with the principles of personal recovery, which emphasize long-term support, relational safety and space for individual processes to unfold (11, 12). Without sufficient time and flexibility, there is little opportunity to co-create realistic coping strategies or foster personal growth. The rigid timeframes in this context, structured around acute care logic, stand in contrast to the more flexible, person-centered approach Medication-Free treatment is supposed to represent (1).

The organizational structure of the ward further reinforced these challenges. Participants described an unclear distribution of roles and responsibilities, which weakened their sense of ownership and professional initiative. According to implementation theory (18), clearly defined roles and sustained leadership engagement are critical elements for implementation success, yet these were absent in the case described. The MHPs’ perception that the treatment model “belonged to management” contributed to passivity, and responsibility was effectively outsourced both upward (to leadership) and laterally (to the music therapist and the sports pedagogue). While activities such as music and sports can contribute positively to the therapeutic environment, assigning core relational responsibilities to peripheral staff may weaken the therapeutic framework necessary to support meaningful psychological change (6).

Moreover, these dynamics appeared to affect patient care. MHPs described how organizational pressures, unclear guidance, and lack of team-based collaboration made it difficult to respond flexibly to patient needs. Despite the rhetoric of shared decision-making and person-centered care (2, 4), the treatment in practice remained heavily influenced by the biomedical model both in structure and culture. This mismatch between ideals and practical conditions suggests that, without fundamental shifts in institutional culture, role empowerment and resource allocation, Medication-Free treatment may remain more symbolic than transformative. In line with implementation research (18), this underscores the necessity of tailoring the implementation effort to the needs of front-line staff, involving them in planning, ensuring consistent leadership and fostering team-based structures.

The findings in this study bring to light a fundamental dilemma for the future of Medication-Free services in mental healthcare. Is it possible to transform existing, medication-centered institutions into milieus that fully embrace recovery-oriented, Medication-Free practices? Or would it be more effective to establish entirely new units, designed from the ground up, with dedicated staff, tailored organizational structures, and a shared understanding of mental health that aligns with non-medical paradigms? The experiences of MHPs in this study suggest that without radical changes in practice, organization and professional culture, attempts to integrate Medication-Free approaches into traditional systems may continue to face resistance, fragmentation and limited impact. This dilemma calls for strategic reflection and bold decisions in future service development.

### Methodological discussion

Conducting a case study was considered appropriate for exploring essential features of implementing Medication-Free services in a milieu therapeutic context. Case study design enables detailed, context-sensitive investigation of complex practices and professional experiences in real-life settings (30, 31). This approach allowed us to capture a situated and contextually embedded understanding of how Medication-Free services are experienced by MHPs. However, local cultural and organizational practices can influence both the statements made by participants and how we interpret them. Such contextual factors should be considered as a reservation for the understanding of the findings. While not intended to produce generalizable findings, the results may be considered transferable to other mental healthcare inpatient settings, particularly in Norwegian and other contexts where similar recovery-oriented treatment ideals are being implemented (37).

The study’s scope was limited to one specific ward, which naturally restricts the breadth of perspectives. However, this narrow focus facilitated a rich exploration of a concrete and operationalized example of Medication-Free treatment in practice, highlighting dynamics that may resonate across similar institutional contexts. As Øvretveit (38) notes, evaluations can only capture selected dimensions of an intervention, and other meaningful outcomes or stakeholder perspectives may remain undiscovered.

A random sample of employees of the ward’s eligible MHPs participated in the study, divided into two focus groups with five participants each. While this sample size may appear limited, qualitative research emphasizes depth over breadth, and thick descriptions were achieved within the scope of the study. The focus group format enabled dialogical exploration and collective reflection among participants, allowing for the articulation of both shared and divergent experiences (37). Nevertheless, it is possible that group dynamics influenced participants’ willingness to voice dissenting or critical perspectives, particularly in a workplace setting.

Our decision to focus on MHPs was deliberate, as they are the primary stakeholders responsible for the therapeutic milieu and the everyday implementation of Medication-Free care. Their perspectives are crucial for understanding both relational and organizational aspects of this treatment model. Including additional professional voices, such as those of psychiatrists, psychologists, music therapists and sports pedagogues, as well as administrative leaders, might have provided further insight into institutional and strategic considerations. However, this would have come at the expense of depth in the MHPs’ accounts. Future studies may benefit from a broader interprofessional perspective to complement these findings. Our results might be seen as an illustration of the potential conflicts when ‘counter-medical’ interventions are introduced in a medicalized structure. They may also highlight issues with the power shift included in the possibility for service users to choose a medication free alternative in settings with hierarchical and medical leadership. Our study was conducted in one ward where all these contradictions were gathered. Future research should study these processes both from the service users’ and the traditional professions’ perspectives. Studies should also focus on how the problems we found have been handled in other situations, from the decisional process to the daily practice.

Researcher positioning is another important consideration. One of the authors was employed at the same department as the participants in the current study during the time of data collection. Although not involved in conducting or in the analyses of the interviews, this proximity may have influenced the framing and discussion of findings. At the same time, the insider perspective may have contributed to greater contextual sensitivity and analytic relevance. In his book, Science of science and reflexivity Bourdieu (39) urge social scientists to not only work reflexively after the research has been launched or when the material has been collected, but also before, when planning the research; a “reflexive reflex” (p 89). Even if it is not possible to fully fulfill Bourdieu’s intention in the framework of this article we have tried to apply some of his ideas. In this research, an important aspect was the context we were to study, but also the context of this context. Norwegian authorities had decided that each region should start a Medication-Free service. Thus, psychiatric services with their bio-medical paradigm had to organize interventions contraire to their own philosophy. We could have chosen to study the implementation of the decision of creating Medication-Free services and how the decisional levels did cope with this paradoxical demand they had to realize, but we started our study when the Medication-Free service already was established. Therefore, we chose to listen to the levels of health professionals in everyday relationships to the service users; “street level bureaucrats” (40). We were conscious that a consequence of our choice was that we missed important parts of the decisional process leading to the positive and critical aspects described by the participants. However, we think that giving voice to these participants was important, even if given a voice does not imply the absence of a critical distance to it. The research team’s mixed professional composition touches another risk that arises from a lack of ‘reflexive reflex’. It was developed to counteract what Bourdieu (39) mention as a “narcissistic reflexivity” (p 89). This kind of reflexivity appears in analysis characterized by the researchers’ preconceptions. The team’s mixed background was also a way to counteract the effect of having an ex-member of the service in the research team.

To enhance the credibility of the study, several steps were taken to ensure trustworthiness in the analytical process. Three of the authors independently conducted thematic analyses of the transcripts, which allowed for analytic triangulation. The preliminary themes were then compared and discussed in detail among the authors until consensus was reached. This process helped reduce individual bias, strengthen interpretive validity, and ensure that the findings were grounded in the data rather than shaped by a single researcher’s perspective. Triangulation through multiple researchers is considered an important strategy to increase credibility in qualitative research (37).

Data were collected in June 2020, approximately two years after the ward began offering Medication-Free services. This represents an early phase in the implementation process, which may help explain some of the uncertainty, lack of structure, and resistance described by participants. Implementation research shows that early-stage efforts are often marked by unclear roles, low organizational readiness and variable staff engagement (19, 23, 41). Moreover, data collection occurred during the first year of the COVID-19 pandemic, a period that may have further disrupted staffing levels, training efforts and overall service stability. These contextual factors should be considered when interpreting and understanding the findings. Nevertheless, the results may contribute to ongoing discussions about how such services can be meaningfully integrated into traditional mental health care systems.

## Conclusion

This study reveals how systemic and relational challenges complicate the implementation of Medication-Free services in traditional psychiatric settings. These findings are situated in a context where national policy mandates shape organizational priorities, and transferability to other health systems will depend on local policy frameworks, resource allocation, and cultural attitudes toward mental health care.

A mismatch between the principles of Medication-Free care and existing organizational structures risks undermining treatment efforts. For such services to succeed, structural changes are needed to support interdisciplinary collaboration, clarify roles and prioritize relational work. Sustainable implementation depends not only on policy, but on everyday practices shaped by the lived realities of both patients and professionals.

Importantly, implementation efforts unfolded during the COVID-19 pandemic, which introduced staffing shortages and resource constraints. These contextual factors likely amplified systemic challenges and should be considered when interpreting the findings.

Implications for practice primarily concern contexts with similar policy mandates and organizational structures. Organizational restructuring that supports interdisciplinary collaboration, distributed leadership and development of relational competence remains critical. Medication-Free services should be rooted in environments allowing for person-centered care, therapeutic continuity during admission and after discharge, and shared decision-making.

Further research should explore how Medication-Free services can be adapted or scaled in diverse health systems and assess the role of contextual factors—such as policy, resources, and workforce stability—in shaping implementation. Evaluating the impact of such changes on patient outcomes, the experiences of MHPs and service quality will be essential. Additionally, developing and testing digital tools to support relational competence and implementation readiness may offer broadly applicable solutions for future practice.

## Data Availability

The raw data supporting the conclusions of this article will be made available by the authors, without undue reservation.
